# Sagace: A web-based search engine for biomedical databases in Japan

**DOI:** 10.1186/1756-0500-5-604

**Published:** 2012-10-31

**Authors:** Mizuki Morita, Yoshinobu Igarashi, Maori Ito, Yi-An Chen, Chioko Nagao, Yuki Sakaguchi, Ryuichi Sakate, Tohru Masui, Kenji Mizuguchi

**Affiliations:** 1 Department of Fundamental Research, National Institute of Biomedical Innovation, 7-6-8 Saito Asagi, Ibaraki, Osaka, Japan; 2 Department of Disease Bioresources Research, National Institute of Biomedical Innovation, 7-6-8 Saito Asagi, Ibaraki, Osaka, Japan

**Keywords:** Search engine, Biomedical data, Biomedical resources, Faceted search, Microdata

## Abstract

**Background:**

In the big data era, biomedical research continues to generate a large amount of data, and the generated information is often stored in a database and made publicly available. Although combining data from multiple databases should accelerate further studies, the current number of life sciences databases is too large to grasp features and contents of each database.

**Findings:**

We have developed Sagace, a web-based search engine that enables users to retrieve information from a range of biological databases (such as gene expression profiles and proteomics data) and biological resource banks (such as mouse models of disease and cell lines). With Sagace, users can search more than 300 databases in Japan. Sagace offers features tailored to biomedical research, including manually tuned ranking, a faceted navigation to refine search results, and rich snippets constructed with retrieved metadata for each database entry.

**Conclusions:**

Sagace will be valuable for experts who are involved in biomedical research and drug development in both academia and industry. Sagace is freely available at http://sagace.nibio.go.jp/en/.

## Findings

Modern biomedical research produces increasing amounts of data, much of which is stored in numerous public databases. (Some of these databases are described in the Database Issue of Nucleic Acids Research each year [1]). As life sciences become ever more data-driven, there is great potential for mining multiple different databases and generating a new knowledge. The sheer number of databases, however, makes data integration a formidable task.

To tackle this issue, the Database Center for Life Science (DBCLS; [2]) and the National Bioscience Database Center (NBDC; [3]) were established in Japan in 2007 and 2011, respectively, with the mandate to archive and integrate Japan’s life sciences databases. In an effort to promote effective data integration, they compiled a database list and developed a framework for distributed search systems, based on which, designated national centers can create domain-specific search websites. The indexes for the selected databases were created by NBDC and other designated national centers, including the National Institute of Biomedical Innovation (NIBIO; [4]).

**Figure 1:**
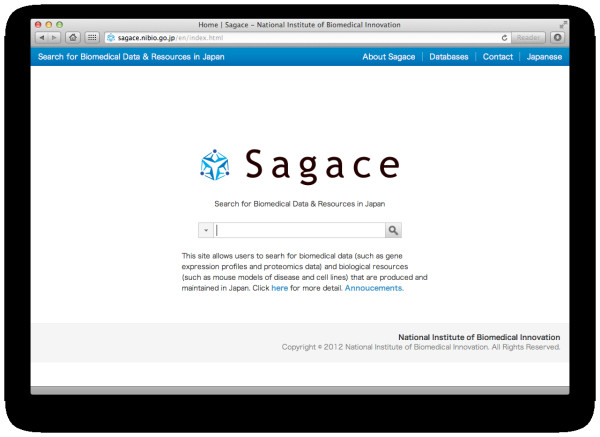
**The top page of Sagace.** Sagace can be freely accessed at http://sagace.nibio.go.jp/en/.

In close collaboration with the DBCLS and the NBDC, we at the NIBIO have developed a search web site called ‘Sagace’ (Figure 1), as a first step towards efficient integration and retrieval of biomedical data from online public databases. This search web site has been customized to search more than 300 biomedical databases in Japan, containing biological data such as gene expression and proteomics data, and biological resources such as mouse disease models and human cultured cells. Our aim is to build a search web site that can assist quick and accurate data retrieval. Here, we describe technical aspects and usage examples of ‘Sagace’.

### Features of Sagace

The core search engine of Sagace is a full-text search system, which searches for user-supplied query terms in all stored documents, similar to current popular search engines such as Google and Yahoo!. However, these general-purpose search engines often fail to retrieve relevant biological data, because, unlike standard web pages, specialist biological databases tend to provide only short natural language descriptions for the individual entries. General-purpose search engines typically rank search results according to the number of matched query terms in each stored document (with adjustments based on the frequency distribution of query terms in the document collection). Therefore, documents with short text are ranked low by standard document retrieval systems. Even if a search engine retrieves entries from a biological database, the user often finds it difficult to judge their relevance, because of the lack of textual information. To address these issues, we implemented three features in Sagace: i) manually assigned weights to the crawled databases for improving the ranking system, ii) a faceted system to refine the search results effectively, and iii) rich snippets to show informative metadata for databases.

First, we have introduced a system that modifies the order of search results according to the weights assigned to individual databases. We have examined manually all the (>300) crawled databases and assigned two different weights. The higher weight has been assigned to the databases that are relevant to human disease and drug discovery. Thus, plant and bacterial databases have tended to be (but not always) assigned the lower weight. We have also assigned the lower weight to many of the reference-type databases such as biological term dictionaries and patent databases, since these databases are likely to be ranked high (irrespective of their content) in the full-text search engine adopted by Sagace (see below). Because the current weights were defined subjectively and thus difficult to evaluate, we plan to optimize the weights by using a more automatic method, for examples, based on the access log data.

**Figure 2:**
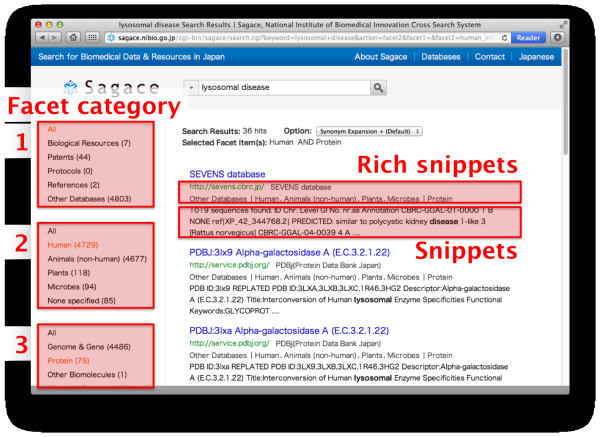
**The search result page of Sagace.** Summary information (in the form of snippets and rich snippets) about each search result helps users judge the relevance of the search result. When the number of search results is large, the users can refine their search results with three types of facet categories on the left column. Combining more than one facet categories refines the search results further. The users can also refine the search results using information in the rich snippets, such as the database name and the species.

Second, to assist in improving search results, we implemented a faceted system. It was reported that users tend to use filters to narrow down the search results and change the queries *after the search*, rather than configuring search engine parameters *before the search*[5]. Therefore, in Sagace, we implemented a faceted system to narrow down the search results [6]. We examined and classified all the crawled databases from three different points of view: 1) the content type (e.g., biological resources, protocols, and references), 2) the species of organism that each database covers, and 3) the level of organization (e.g., genome and gene, cell and tissue, and organism). Using these three categories, users can apply multiple filters and refine their search results effectively. In Sagace, users can use the three facet categories displayed on the left of the search results (Figure 2).

Last, we implemented in Sagace snippets and rich snippets, the few lines of text that appear under each search result, to provide users with condensed information about the retrieved page (Figure 2). Generally, the snippet for a page includes its summary description, while the rich snippet shows page metadata. To create rich snippets for entries from biological databases, Sagace retrieves the metadata for each database entry page, if they are stored in the microdata format [7].

Recently, several metadata formats for web pages have been proposed, such as RDFa [8], microformats [9], and microdata [7]. Among these, we have decided to use microdata, because it was adopted by schema.org [10], a collection of schemas for structured data markup on web pages, proposed by three major search engines (Google, Yahoo! and Microsoft) [11-13]. The vocabulary on schema.org provides an unambiguous description of data attributes such as the name of the item, the URL, the creation date, the last modification date, keywords and the provider name. However, there was no adequate vocabulary set on schema.org for biological databases and therefore, we have proposed an extension to the schema.org vocabulary [14]. Attributes specific to biological database entries can now be described, including the entry identifier, the database name to which the entry belongs and the taxonomy identifier of the entry. When our extension is officially approved and as long as the database providers specify these attributes in the given format, search engine crawlers will recognize the meaning of these attributes and retrieve the relevant information. As an example, we introduce searching with an entry identifier. By adding a “[id]” tag after the query term, users can directly search for a specific database entry with the specified identifier. Currently, only a few of the crawled databases offer the entry ID field but since the schema.org extension that we propose contains “entryID” in its data structure, this approach will work more efficiently over time when the schema.org extension becomes widely adopted by database providers.

Currently, only the Japanese Collection of Research Bioresources (JCRB) Cell Bank [15] has officially employed our proposed vocabulary but we expect the importance of our proposal to be recognized more widely, since providing structured information is the best way to organize and integrate a large number of databases. With more databases adopting this vocabulary set, metadata for biological databases should provide not only better search experiences but also novel applications. For instance, by collecting microdata for biological databases in a systematic manner, a catalogue of biological databases can be constructed automatically. It would also be possible to develop script libraries that utilize microdata information, such as those for Google Maps [16]. For example, a library can be written to obtain the species information from a database entry and display the corresponding ‘Taxonomy icon’ [17], which is a graphical image representing each species.

Sagace is similar to other cross-database search systems such as Entrez [18,19] at the National Center for Biotechnology Information (NCBI) and EB-eye [20,21] at the European Bioinformatics Institute (EBI). While Entrez allows users to search not only indexed text but also any value in the data (including sequences and numerical counts), EB-eye focuses on an indexed collection of selected textual content (such as gene names and descriptions). In this sense, Sagace, as a textual search engine, is more similar to EB-eye than Entrez. However, unlike Entrez and EB-eye, which navigate through the databases hosted by NCBI and EBI, respectively, Sagace searches a wide collection of biomedical database on the web (including small and specialist databases). This characteristic makes the range of Sagace users more diverse than those of the two other search engines. It requires the search interface to meet wider demands of users and to adapt to unscheduled format changes in the crawled databases. It is these factors, while making search results of Sagace less structured than those of Entrez and EB-eye, that motivated us to propose and promote the schema.org extension for biological databases; we aim to produce some sort of structured results with a minimal effort from database providers. Besides, the faceted search allows to narrow down the search results from various aspects, and the rich snippets help users to grasp quickly a summary of each entry.

### Implementation

We employed Hyper Estraier [22] as a core search engine to construct our search system. Hyper Estraier is an open source full-text search engine equipped with all basic features of full-text search as well as multilingual support. Hyper Estraier also utilizes a Peer-to-Peer (P2P) distributed search technology to build large-scale search applications. Multiple organizations can take charge of crawling different databases, and the resulting inverted index files can be shared. We collaborate with the NBDC and maintain our crawling system together. The NBDC sets up its own search engine [23], and both our search engines access the common inverted index files on the fly. Sagace, however, assigns weights to a selected subset of the databases, as described above, and thus, search results can be different between the two search engines.

### Example usage

We present two usage examples of Sagace: one to collect information about a specific gene, and the other to find distributors of particular cultured cells.

In 2001, Eisenberg et al. identified GNE (the gene encoding bifunctional UDP-N-acetylglucosamine 2-epimerase/N-acetylmannosamine kinase) as the causal gene for Distal Myopathy with Rimmed Vacuoles [24]. As shown in the gene product name, GNE is a fusion gene of two enzymes and has two distinct functions. Querying Sagace with the full name of this gene product “bifunctional UDP-N-acetylglucosamine 2-epimerase/N-acetylmannosamine kinase” produces around 40 search results. To restrict search results to only those from gene-related databases, users can click on the ‘Genome & Gene’ facet on the left of the result page. Among the restricted search results, a hit to a KEGG [25] entry shows that this gene is the causal gene for another disease named ‘Sialuria’. Another hit to FLJ Human cDNA Database [26] indicates that the gene locates on human chromosome 9. Moreover, other pages lead the user to multiple three-dimensional structures of human N-acetylmannosamine kinases in PDBj [27], known single nucleotide polymorphisms for the queried gene in GeMDBJ [28], and protein-protein interaction information in the Genome Network Project [29].

A second example is to find specific Induced Pluripotent Stem (iPS) cells [30] for research purposes. A number of search results will be returned by a query with “iPS”. Adding a query term “lung” will reduce the search results dramatically. To narrow down the search results further, select ‘Biological Resource’ in the facet categories at the upper left column of the page. If necessary, the list may be narrowed down further by selecting ‘Human’ in the facet categories at the lower left column. From the refined list of search results, the user can easily find the required cell type (e.g., human lung fibroblast-derived iPS cells), along with the distributor details.

## Availability and requirements

**Project name**: Sagace.

**Project home page**: http://sagace.nibio.go.jp/en/.

**Computer system requirements**: Any operating system with any modern web browser.

**Any restrictions to use by non**-**academics**: None.

## Abbreviations

DBCLS: Database Center for Life Science; GNE: The gene encoding bifunctional UDP-N-acetylglucosamine 2-epimerase/N-acetylmannosamine kinase; iPS cell: Induced Pluripotent Stem cell; NBDC: National Bioscience Database Center; NIBIO: National Institute of Biomedical Innovation.

## Competing interests

The authors declare that they have no competing interests.

## Authors’ contributions

MM designed the web interface. MM, YI, YC, CN, RS, and KM selected and ranked the crawled databases. RS, YS, and TM provided data for the NIBIO databases. All authors tested and contributed to the user interface. YI, MI and KM participated in developing the website. MM and YI wrote the manuscript. KM supervised the project and assisted in the editing of the manuscript. All authors read and approved the submitted manuscript.
